# Female genital mutilation/cutting in sudan and subsequent pelvic floor dysfunction

**DOI:** 10.1186/s12905-021-01576-y

**Published:** 2021-12-28

**Authors:** Özer Birge, Aliye Nigar Serin, Mehmet Sait Bakır

**Affiliations:** 1 Department of Gynecology and Obstetrics, Nyala Sudan Turkey Training and Research Hospital, West Alezza District Southern, 63311 Nyala, Darfur Sudan; 2 Department of Gynecology and Obstetrics, Karamanoglu Mehmet Bey University, Karaman, Turkey; 3 Department of Gynecology and Obstetrics, Akdeniz University, Antalya, Turkey

**Keywords:** Assisted vaginal delivery, Female genital mutilition/cutting, Hydronephrosis, Pelvic floor, Pelvic organ prolapse, Urinary incontinence

## Abstract

**Background:**

We aimed to evaluate the socio-demographic characteristics of women with female genital mutilation/cutting (FGM/C) and the results of FGM/C due to pelvic floor dysfunction.

**Results:**

The prevalence of FGM/C was 87.2% in Sudan and Type 3 (50.4%) was the most prevalent, followed by Type 2 (35%) and Type 1 (8.5%). In the multinominal logistic regression analysis performed to show the effect of FGM/C on pelvic organ prolapse (POP), it was observed that FGM/C frequency in POP group 2 was statistically similar when POP group 1 was taken as reference category. In the evaluation for symptomatic POP (POP group 3), risk of developing POP in patients without FGM/C was significantly lower than patients with type 3 FGM/C with a rate of 82.9% (OR(odds ratio): 0.171 (p: 0.002), (Confidence Interval (CI) %95; 0.058–0.511). Risk of developing POP rate in patients with type 1 FGM/C was 75% (OR:0.250 (p: 0.005), CI %95; 0.094–0.666) and in patients with type 2 FGM/C was 78.4% (OR:0.216 (p: 0.0001), CI%95; 0.115–0.406). In the multinominal logistic regression analysis including other variables affecting POP, when group 1 was taken as the reference category, it was found that the possibility of developing mild POP (group 2) decreased in FGM/C type 1 and 2 compared to FGM/C type 3 but it was not statistically significant. However, the evaluation for the symptomatic POP group showed up a significantly lower risk of developing POP in patients with type 2 FGM/C compared to patients with type 3 FGM/C, with a rate of 58.4%. (OR:0.419 (p: 0.016), CI%95; 0.206–0.851) (Table 3). In addition, older age was found to be significant risk factor for increasing symptomatic POP (p: 0.003).

**Conclusions:**

Type 2 and 3 FGM/C continues to be an important health problem in terms of complications that may develop in advanced ages as well as many short-term complications as a result of mechanical or physiological deterioration of the female genital anatomy.

## Background

The World Health Organization (WHO) has described female genital mutilation/cutting (FGM/C) as any procedure that involves the removal of the external female genitalia partially or totally or any injury to the female genital organs for cultural or any other non-medical reasons [1]. The word “mutilation” emphasizes the violence of the practice [2]. Four different types of FGM/C have been defined by WHO based on which genital tissue has been removed: Type I (Sunna, mild) refers to partial or total removal of the preputium with or without the clitoris; Type 2 (excision, moderate) refers to clitoridectomy and partial or total excision of the labia minora; Type 3 (infibulation, severe) refers to removal of the complete external genitalia and narrowing of the vaginal opening to a small orifice; and Type 4 (unclassified) refers to other harmful procedures to the female genitalia, such as piercing and pricking [1, 2].

Although the worldwide prevalence of FGM/C is unknown, it has been applied to more than 200 million women in 30 countries in Africa, the Middle East, and Asia and is an ongoing practice [1]. FGM/C has also been reported to take place in other countries, including the United States, Spain, and countries in South America, due to widespread migration [2] and is prevelant in many Muslim countries, even though neither Islam nor any other religion requited FGM/C [3–5]. The prevalence rates of FGM/C vary considerably in African countries; the highest rate of FGM/C was reported in Somalia (98%) and Guinea (97%) [6]. In a recent survey study of 21,947 Sudanese women, the prevalence of FGM/C was 89% [7]. The type of procedure performed also varies with ethnicity [2].

FGM/C is thought to be a tribal tradition or an Islamic imperative, and several studies have reported the reasons that a girl might undergo FGM/C include providing her with an honorable social life, preserving her virginity, and allowing her to become a mature woman for a safe marriage [7, 8]. Moreover, it is believed that FGM/C provides hygiene and makes women cleaner and more beautiful, and FGM/C-prevalent societies consider it a prerequisite for marriage [9]. In reality, FGM/C is a violation of basic human rights, and WHO and UNICEF stand against FGM/C based on its negative impact on women’s health [10].

FGM/C is widely performed in Sudan by midwives on girls between the ages of 6 and 12 without any anesthesia or the use of antibiotics [11]. It is well-known that FGM/C is a harmful procedure that causes many short- and long-term health consequences, depending on the type of FGM/C. The short-term physical complications include severe pain, swelling of the genital tissue, infection or tetanus due to unhygienic conditions and unsterilized instruments, fever, acute haemorrhage and related haemorrhagic shock and death, failure of the wound to heal, acute urine retention and related urinary tract infection, damage to the adjacent tissue of the vagina, urethra and rectum, fracture or dislocation of the femur or humerus, and serious psychosocial and sexual function impairments [1, 2]. The long-term consequences include recurrent/chronic urinary tract, pelvic, and vaginal infections, painful urination, incontinence, female sexual dysfunctions (dyspareunia, reduced sexual sensitivity, female orgasmic disorders, vaginismus, vaginal penetration difficulties), menstrual problems (dysmenorrhoea, haematocolpos), infertility, keloid scarring, epidermoid inclusion cyst and neuroma of the clitoris, abscesses on the vulva, vesico-vaginal or recto-vaginal fistulae, childbirth complications (post-partum haemorrhage, deep tearing of the perineum, prolonged and obstructed labour, fistula, inertia or rupture of the uterus, increased risk of emergency caesarean section, and maternal death), perinatal risks (the need to resuscitate, stillbirth), pelvic organ prolapse, the need for later surgeries (deinfibulation, clitoral reconstruction, urogynecological procedures), and psychological consequences (depression, anxiety, post-traumatic stress disorder) [1–4, 12–16]. All these risks increase with the severity of the FGM/C procedure [16]. Deinfibulation and clitoral reconstruction allow intercourse, facilitate childbirth, and improve both sexual function and genital anatomy [5, 17].

As the severity of FGM/C increases, pelvic floor weakness and pelvic floor disorders are more likely to occur in the long-term due to procedural injury of pelvic tissues and the consequenses of the FGM/C procedure, including difficult deliveries. Loss of pelvic floor support causes various degrees of pelvic organ prolapse, incontinence, anatomical distortion of the lower urinary tract, ureteral kinking and, finally, hydroureteronephrosis. Although multiple studies have investigated the psychological and obstetric consequenses of FGM/C, only a few studies have been conducted on urogynecological outcomes [13, 18, 19].

No previous studies have evaluated the association between the stage of pelvic organ prolapse and FGM/C type, which was expected to be clarified by this study, whose aim was to investigate the impact of different types of FGM/C on pelvic floor disorders such as pelvic organ prolapse and related hydroureteronephrosis and incontinence.

## Material and methods

4320 women who applied to the Sudan Nyala Turkish Hospital Gynecology and Obstetrics outpatient clinic between January 2018 and January 2019 were asked about any prolapsus of the genital organs, and 528 women with pelvic organ prolapsus were included in the study and classified according to the Baden–Walker halfway scoring system. This study was conducted retrospectively at a single center and approved by the Ethical Review Committee of Sudan Nyala Turkish Hospital (Ethics Committee decision dated June 26, 2018, no. 45/4743). Pregnant women, women under 18 years old, women with a history of prior pelvic surgery or other treatments such as using a pessary for POP, and women with FGM/C type 4 were excluded from the study.

The Baden–Walker halfway systems consists of four grades: grade 0 = no prolapse, grade 1 = halfway to the hymen, grade 2 = to the hymen, grade 3 = halfway past the hymen, grade 4 = maximum descent. In our study, patients without prolapse (grade 0) were group 1; patients with grade 1 and 2 POP who were asymptomatic (without urinary incontinence, dyspareunia, dysuria, or frequent urinary tract infection) were group 2, and severely symptomatic cases (grade 3 and 4 POP) were group 3.

Patients with FGM/C were classified into three groups according to the WHO’s typing of FGM/C, and 32 patients without FGM/C were included as a control group. A total of 528 patients in four groups were included in the study, and variables were compared. Demographic and clinical characteristics of the patients, including age, parity, type of birth, comorbidities, incontinance, smoking, region of residence, job, educational status, and menopausal status were recorded using patient files and the hospital system. The body mass index (BMI) of patients was calculated in kg / m^2^ and grouped as < 25 and ≥ 25.

For descriptive statistics, the mean, standard deviation, median, min–max values ​​and frequencies were used, depending on whether there was a normal distribution or not. Statistical significance between categorical variables was determined by Chi-Square (χ2) test. Normal distribution for numerical data was made using the Kolmogorov–Smirnov test. For numerical data, parametric or non-parametric tests were used according to the normal distribution state. Kruskal–Wallis test was used to analyze the age difference between the groups. Univariate Multinomial logistic regression analysis was used for the relationship between FGM/C and pelvic organ prolapse, taking Group 1 (non-POP cases) as the reference category. Risk factors that were significantly associated with POP as a result of the univariate analysis (p value less than 0.05) were included in the multivariate multinominal logistic regression analysis. Multivariate multinominal logistic regression analysis was performed by adding variables such as, residence (rural, urban), age, job (yes, no), parity (yes, no), smoking (yes, no), BMI (< 25, ≥ 25), menopausal status (yes, no). The data was analyzed by using Statistical Package for the Social Sciences (SPSS)-23.0 program. P values in all tests are two-sided, and p-value less than 0.05 was considered to be statistically significant.

## Results

The number of women with female genital mutilation/cutting (FGM/C) was 3767 out of 4320 women. The prevalence of FGM/C among all women in Sudan was 87.2%. The prevalence of FGM/C among 528 women with pelvic floor dysfunction was 87%. The demographic and clinical characteristics of the patients were shown in Table 1. 496 patients with FGM/C were divided into 3 groups according to the WHO’s classification; 45 patients (8.5%) were FGM/C Type 1, 185 patients (35%) were Type 2 FGM/C, 266 patients (50.4%) were Type 3 FGM/C. 32 (6.1%) patients with no cutting were determined as the control group. Regarding the type of FGM/C performed, Type 3 was the most prevalent, followed by Type 2 and Type 1.

**Table 1:** Demographic and clinical features of POP patients

		No POP (Group 1) (n: 69)	Mild POP (Group 2) (n: 230)	Severe POP (Group 3) (n: 229)	Total (n: 528)	*P *value
Age (median/min–max)		42 (32–65)	44 (31–88)	49 [30–91]		***0***.***001***^**a**^
BMI (kg/m^2^)	< 25	25 (4,7%)	74 (14%)	29 (5.5%)	128 (24.2%)	***0.001***^**b**^
	≥ 25	44 (8.3%)	156 (29.5%)	200 (37.9%)	400 (75.8%)	
Job	No	43 (8.1%)	135 (25.6%)	93 (17.6%)	271 (51.3%)	***0.001***^**b**^
	Yes	26 (4.9%)	95 (18%)	136 (25.8%)	257 (48.7%)	
Parity	No	7 (1.3%)	15 (2.8%)	6 (1.1%)	28 (5.3%)	***0.027***^**b**^
	Yes	62 (11.7%)	215 (40.7%)	223 (42.2%)	500 (94.7%)	
Urinary ıncontinence	No	29 (5.5%)	122 (23.1%)	26 (4.9%)	177 (33.5%)	***0.001***^**b**^
	Yes	40 (7.6%)	108 (20.5%)	203 (38.4%)	351 (66.5%)	
HUN	No	41 (7.8%)	109 (20.6%)	34 (6.4%)	184 (34.8%)	***0.001***^**b**^
	Yes	28 (5.3%)	121 (22.9%)	195 (36.9%)	344 (65.2%)	
Smoking	No	68 (12.9%)	214 (40.5%)	205 (38.8%)	487 (92.2%)	***0.041***^**b**^
	Yes	1 (0.2%)	16 (3%)	24 (4.5%)	41 (7.8%)	
Assisted vaginal delivery	No	45 (8.5%)	163 (30.9%)	62 (11.7%)	270 (51.1%)	***0.001***^**b**^
	Yes	24 (4.5%)	67 (12.7%)	167 (31.6%)	258 (48.9%)	
Delivery method	No	7 (1.3%)	15 (2.8%)	6 (1.1%)	28 (5.3%)	***0.001***^**b**^
	VD	52 (9.8%)	170 (32.2%)	201 (38.1%)	423 (80.1%)	
	C/S	10 (1.9%)	45 (8.5%)	22 (4.2%)	77 (14.6%)	
Parity	No	7 (1.3%)	15 (2.8%)	6 (1.1%)	28 (5.3%)	***0.001***^**b**^
	1	22 (4.2%)	65 (12.3%)	30 (5.7%)	117 (22.2%)	
	02-May	38 (7.2%)	138 (26.1%)	95 (18%)	271 (51.3%)	
	≥ 5	2 (0.4%)	12 (2.3%)	98 (18.6%)	112 (21.2%)	
Residence	Rural	43 (8.1%)	153 (29%)	165 (31.3%)	361 (68.4%)	0.227^b^
	Urban	26 (4.9%)	77 (14.6%)	64 (12.1%)	167 (31.6%)	
Educational status	No	32 (6.1%)	112 (21.2%)	125 (23.7%)	269 (50.9%)	0.324^b^
	Read and write	37 (7%)	118 (22.3%)	104 (19.7%)	259 (49.1%)	
Menopausal status	No	54 (10.2%)	151 (28.6%)	120 (22.7%)	325 (61.6%)	***0.001***^**b**^
	Yes	15 (2.8%)	79 (15%)	109 (20.6%)	203 (38.4%)	
Baseline comorbidity	No	68 (12.9%)	219 (41.5%)	177 (33.5%)	464 (87.9%)	***0.001***^**b**^
	Yes	1 (0.2%)	11 (2.1%)	52 (9.9%)	64 (12.2%)	
FGM/C type	No	7 (1.3%)	16 (3%)	9 (1.7%)	32 (6.1%)	***0.001***^**b**^
	Type 1	8 (1.5%)	22 (4.1%)	15 (6.6%)	45 (8.5%)	
	Type 2	34 (6.4%)	96 (18.2%)	55 (10.4%)	185 (35%)	
	Type 3	20 (3.8%)	96 (18.2%)	150 (28.4%)	266 (50.4%)	

Statistically significant *p* values are numbered in bolditalics

POP: Pelvik organ prolapsus. BMI: body mass ındex., HUN: hydroureteronephrosis, VD: vaginal delivery, C/S: cesarean section, FGM/C: female genital mutilation/cutting

^a^Kruskal-wallis

^b^Chi-square

The median age of symptomatic patients (group 3) was 49 (min: 30-max: 91), and it was significantly higher than the other groups (p: 0.001).

The number of patients living in rural areas was higher in all 3 groups, the highest rates of POP were among housewives and unemployed women.

There was a significant difference between the groups in terms of BMI and menopausal status (sırasıyla p: 0.001 ve p: 0.001). It was observed that symptomatic POP was more common in patients who gave birth (p: 0.027) and as the parity increased, the frequency of POP increased (p: 0.001). Approximately 423 of the patients had a normal vaginal delivery (p: 0.001), 258 of them had a history of assisted vaginal delivery (p: 0.001). 487 of our patients in the study were non-smokers. The frequency of incontinence increased among the groups as the degree of pelvic organ prolapse increased (p: 0.001). The frequency of hydronephrosis in group 3 POP cases was significantly different from the other groups (p: 0.001). No statistical difference was found between the POP groups in terms of the patients' residence and education level (p: 0.227, p: 0.324; respectively). It was observed that Type 3 FGM/C was performed in 266 patients, and it was the largest group in group 3 patients with a rate of 28.4% (p: 0.001).

In the multinominal logistic regression analysis performed to show the effect of FGM/C on pelvic organ prolapse, it was observed that FGM/C frequency in group 2 was not statistically different when the reference category was taken as group 1. It was observed that symptomatic POP (group 3) rate statistically significantly decreased in other types compared to Type 3 FGM/C (respectively, No-FGM/C OR(odds ratio): 0.171 (p: 0.002), (Confidence Interval (CI) %95; 0.058–0.511), FGM/C tip 1 OR:0.250 (p: 0.005), CI %95; 0.094–0.666), FGM/C tip 2 (OR:0.216 (p: 0.0001), CI%95; 0.115–0.406) (Table 2). In the evaluation for symptomatic POP (POP group 3), risk of developing POP in patients without FGM/C was significantly lower than patients with type 3 FGM/C with a rate of 82.9% (OR(odds ratio): 0.171 (p: 0.002), (Confidence Interval (CI) %95; 0.058–0.511). Risk of developing POP rate in patients with type 1 FGM/C was 75% (OR:0.250 (p: 0.005), CI %95; 0.094–0.666) and in patients with type 2 FGM/C was 78.4% (OR:0.216 (p: 0.0001), CI%95; 0.115–0.406) (Table 2).

**Table 2:** Univariate multinomial logistic regression analysis of the relationship between FGM/C types and POP

		Comparison between group 1 POP and group 2 POP				Comparison between group 1 POP and group 3 POP			
		Unadjusted OR	CI %95		*P *value	Unadjusted OR	CI %95		*P *value
			Lower	Upper			Lower	Upper	
FGM/C status	No FGM/C	0.476	0.173	1.308	*0.150*	0.171	0.058	0.511	***0.002***
	FGM/C type 1	0.573	0.222	1.469	0.246	0.250	0.094	0.666	***0.005***
	FGM/C type 2	0.588	0.316	1.094	0.094	0.216	0.115	0.406	***0.0001***
	FGM/C type 3	1				1			

Statistically significant *p* values are numbered in italics and bolditalics

POP: Pelvik organ prolapsus, FGM/C: Female genital mutilation/Cutting, The reference category is: Group 1

In the multinominal logistic regression analysis performed by including other variables affecting POP when group 1 was taken as the reference category, we found that the possibility of developing mild POP (group 2) decreased in FGM/C type 1 and 2 compared to FGM/C type 3 but it was not statistically significant. However, we found that the effect of type 2 FGM/C on severe symptomatic POP was statistically significantly lower than FGM/C type 3. (OR:0.419 (p: 0.016), CI%95; 0.206–0.851) (Table 3). However, the evaluation for symptomatic POP showed up a significantly lower risk of developing POP in patients with type 2 FGM/C at a rate of 58.4%, compared to type 3 FGM/C (OR:0.419 (p: 0.016), CI%95; 0.206–0.851). In addition, older age was found to be significant risk factor for increasing symptomatic POP (p: 0.003) (Table 3).

**Table 3:** Relationship between multivariate multinominal logistic analysis and POP

		Comparison between group 1 POP and group 2 POP				Comparison between group 1 POP and group 3 POP			
		Adjusted OR	CI %95		*P *value	Adjusted OR	CI %95		*P *value
			Lower	Upper			Lower	Upper	
Age (year)	contious	1.01	0.98	1.04	0.451	1.054	1.018	1.092	***0.003***
BMI (Kg/m^2^)	BMI < 25	1.32	0.63	2.79	0.453	0.442	0.178	1.098	*0.079*
	BMI ≥ 25	1				1			
Smoking	No	0.202	0.021	1.571	0.127	0.177	0.023	1.387	0.099
	Yes	1				1			
Menopause status	No	0.595	0.293	1.214	0.154	0.857	0.404	1.819	0.688
	Yes	1				1			
Parity	No	0.634	0.212	1.94	0.403	0.425	0.126	1.552	0.195
	Yes	1				1			
Live in	Rural	1.19	0.663	2.125	0.556	1.161	0.628	2.145	*0.634*
	Urban	1				1			
Job	No	1.08	0.569	1.781	0.978	0.564	0.312	1.02	0.058
	Yes	1				1	1.01	62.2	
FGM/C status	No FGM/C	0.599	0.178	2.02	*0.409*	0.851	0.216	3.349	0.817
	FGM/C type 1	0.643	0.193	2.145	0.473	1.675	0.432	6.503	0.456
	FGM/C type 2	0.671	0.337	1.334	0.255	0.419	0.206	0.851	***0.016***
	FGM/C type 3	1				1			

Statistically significant *p* values are numbered in italics and bolditalics

POP: Pelvik organ prolapsus, FGM/C: female genital mutilation/Cutting, BMI: body mass ındex, SE: standart error, B: coefficient

Adjusted odds ratio: was used for; age, job (yes, no), parity (yes, no), smoking (yes, no), BMI (< 25, ≥ 25), Menopausal Status (yes,no), Residence (rural, urban), The reference category is: Group 1

## Discussion

This presented study signified that the incidence of symptomatic POP (group 3) increases as the severity of FGM/C increases. It was observed that development of symptomatic POP (group 3) in patients with type 3 FGM/C was approximately 17 times higher than in patients without FGM/C and type 3 FGM/C caused symptomatic POP approximately 2.4 times more than type 2 FGM/C. Our study revealed that FGM/C had a significant relation with symptomatic POP.

Severe FGM/C (especially type 3), which is performed at an early age before the development of the genital organs, disrupts the mechanical structure and dynamism of the endopelvic fascia and causes anatomical defects. Also frequent and chronic infections play an important role in the formation of POP. In addition, factors that impaired the support of the pelvic endopelvic fascia such as delivery (operative vaginal delivery), malnutrition and menopause are known to cause POP. Therefore, it is a logical approach to consider FGM/C has a strong impact on developing POP in the African population, which practices at the juvenile period (most often FGM/C is performed in the 2–8 age range) traumatically to the genital area and causes psychological effects.

Thanks to the recent effective measures of all these international organizations, practicing severe FGM/C types such as type 2 and 3 have been reduced in the societies by the awareness of the harms of this practice, especially in the new generation of young women. It was not surprised that type 3 FGM/C patients in our study were older and had more symptomatic POP. POP, and related complications are seen lower especially in these areas where lower FGM/C ratios in younger age women, more simple excision methods such as type 1 are used and pelvic anatomy is exposed to less trauma.

Advanced invasive procedure carries out severe complications, so type 3 is the most associated type with complications.

This presented study was done in a FGM prevalent country, Sudan, and was designed specifically to investigate the relation between different types of FGM and pelvic floor dysfunction. The median age of participants was 49 years (min:30-max:91) in the study and higher than other studies on FGM/C [3, 16], because it was a study conducted among women with pelvic floor disorders. If we consider the types according to age in our study, we realized that the new generation prefers the less complicated Type of FGM/C due to awareness of the practice. Older age was associated with increased FGM/C rates and more complicated types of FGM/C (Type 2, 3) [21, 22]. Consistently in our study 70 years and older women had Type 3 FGM/C most frequently. The valid reason for the higher frequency of FGM/C in the older population is almost all women had to embrace female circumcision in the past and it was difficult or impossible to come across a woman who had not practiced. However, FGM/C had declined among youth, possibly due to human rights and legal protection, and even due to imposed prison sentences. FGM is now performed in secrecy in some communities or none at all.

Our study pointed that FGM/C was more prevalent among women living in rural areas than women living in urban, in accordance with the literature [7, 23]. As shown in many studies we found that non-employed and non-educated women were more likely to have undergone FGM/C so the significance of women's education to eradicate the practice of FGM/C is obvious [3, 7, 14, 24, 25]. FGM/C practice rates decreased after the introduction of national training programs, and the availability of guidelines for FGM/C management for healthcare professionals and the general population.

The relationship between FGM/C type and delivery outcomes were statistically significant in our study. In accordance with various studies, women with FGM/C Type 3 were significantly more likely to undergo cesarean section (C/S) with a rate 33.8%, followed by Type 2 (10.8%) and Type 1 did not increase the risk for C/S. Wuest et al. reported higher risk for emergency C/S and deep vaginal tears in circumcised women, WHO reported significantly higher C/S and episiotomy rates among women with type 2 and 3 FGM/C and type 1 FGM/C was ineffective on C/S rates [16, 26]. Only a study declared that FGM/C had no risk for delivery except perineal tearing [27]. Spontaneous vaginal delivery was most frequent with a range of 53.1% in no cutting group. Among assisted vaginal deliveried women, episiotomy with or without vacuum or forceps was highly prevalent in women with FGM/C than women without FGM/C. Episiotomy without vacuum or forceps was most common in FGM/C Type 1 group (62.2%), and episiotomy with vacuum or forceps was most common in FGM/C Type 3 group (13.1%). These findings were supported by Yassin et al. with an episiotomy rate of 76.5% [20]. FGM/C is usually performed in girls younger than 10 years old and even the least invasive type causes varying amounts of scar formation. The presence of this less elastic scar tissue causes varying degrees of perineal and vaginal tears during childbirth. Even, Birge et al. have presented a large epidermal inclusion cyst of the clitoris, an intensive scarring mass, in a woman with type 3 FGM/C, blocking urination and sexual functions due to genital anatomical disruption caused by repetitive episiotomies and deinfibulations [28]. Complications of FGM/C are ranging from prolonged labor, assisted delivery, postpartum hemorrhage, difficulty in urination, urinary tract infections, hydronephrosis, kidney failure, urogenital fıstula to maternal and infant death. As a result, prolapse and related complications increase due to defects on the muscles and fascia of the genitalia and pelvic floor, after the deterioration of the genital anatomy and complicated deliveries. Sudan is still one of the highest prevalent country of maternal mortality (311/100000) in the world according to report of WHO in 2015 [29].

Although many studies have been conducted on sexual, physical and obstetric complications, and survey studies have been conducted on the difficulties and reasons of women mutilation experience and practice [3, 15], data about consequences of different types of FGM/C on urogynecological problems such as incontinence is scarce. In a few recent study evaluating urogynecological problems, FGM/C related lower urinary tract problems have been suggested as urgency, urinary retension and urinary incontinence [13, 30]. Incontinence was mostly observed in type 3 FGM/C in our study, followed by type 2, and the most common type was mixt type incontinence. No cutting and type 1 FGM/C was unrelated with incontinence. Nerve damage and loss of strength-injury to the pelvic floor muscles play role of developing incontinence.

Regardless of the type, it is understood that FGM/C is significantly associated with POP. We revealed that type 3 FGM/C, which is the most invasive and hard procedure of FGM/C, is the most related type with pelvic organ prolapsus and related hydronephrosis and incontinence. FGM/C complications are based on damage to pelvic floor muscles and nerves. So to speak, FGM/C is a deliberated pelvic floor injury procedure. When FGM/C-related difficult deliveries and other risk factors of losing pelvic floor support are added to this, pelvic floor dysfunction is inevitable. Weakness of the pelvic floor muscles due to neuropathic damage or mechanical muscular damage causes pelvic organ prolapse and / or dysfunction. This study is one of the pioneering studies investigating the effect of FGM/C types on pelvic floor disorders such as POP and incontinence. However, the relationship between POP and FGM/C decreases when counfounding variables are added to the model. This result proves that we should not ignore the fact that there are many factors affecting POP. But the thought of the relationship between FGM/C and POP would be logical. Hence, it will be more beneficial to design detailed research on this issue.

## Conclusion

In conclusion, despite the anti-circumcision laws and all preventive efforts of the World Health Organization, UNICEF and many local non-governmental organizations, FGM/C still continues at a high rate all over the world. Especially type 2 and 3 FGM/C continues to be an important health problem in terms of complications that may develop in advanced ages as well as many short-term complications as a result of mechanical or physiological deterioration of the female genital anatomy.

## Data Availability

The datasets used and/or analysed during the current study are available from the corresponding author on reasonable request.
